# Specific and Sensitive Detection of Tartrazine on the Electrochemical Interface of a Molecularly Imprinted Polydopamine-Coated PtCo Nanoalloy on Graphene Oxide

**DOI:** 10.3390/bios12050326

**Published:** 2022-05-11

**Authors:** Shuwen Cheng, Danyao Tang, Yi Zhang, Libin Xu, Kunping Liu, Kejing Huang, Zhengzhi Yin

**Affiliations:** 1 College of Biological, Chemical Sciences and Engineering, Jiaxing University, Jiaxing 314001, China; 18268501447@139.com (S.C.); tdy15757933465@163.com (D.T.); 18595672376@163.com (Y.Z.); 13736811542@139.com (L.X.); 2 College of Chemical Engineering, Zhejiang University of Technology, Hangzhou 310014, China; 3 Key Laboratory of Medicinal and Edible Plants Resources Development of Sichuan Education Department, Sichuan Industrial Institute of Antibiotics, Chengdu University, Chengdu 610106, China; liukunping@cdu.edu.cn; 4 China Key Laboratory of Chemistry and Engineering of Forest Products, Guangxi Key Laboratory of Chemistry and Engineering of Forest Products, Key Laboratory of Guangxi Colleges and Universities for Food Safety and Pharmaceutical Analytical Chemistry, School of Chemistry and Chemical and Engineering, Guangxi University for Nationalities, Nanning 530008, China

**Keywords:** platinum cobalt nanoalloy, molecularly imprinted polydopamine, graphene oxide, tartrazine, electrochemical sensor

## Abstract

A novel electrochemical sensor designed to recognize and detect tartrazine (TZ) was constructed based on a molecularly imprinted polydopamine (MIPDA)-coated nanocomposite of platinum cobalt (PtCo) nanoalloy-functionalized graphene oxide (GO). The nanocomposites were characterized and the TZ electrochemical detection performance of the sensor and various reference electrodes was investigated. Interestingly, the synergistic effect of the strong electrocatalytic activity of the PtCo nanoalloy-decorated GO and the high TZ recognition ability of the imprinted cavities of the MIPDA coating resulted in a large and specific response to TZ. Under the optimized conditions, the sensor displayed linear response ranges of 0.003–0.180 and 0.180–3.950 µM, and its detection limit was 1.1 nM (S/N = 3). The electrochemical sensor displayed high anti-interference ability, good stability, and adequate reproducibility, and was successfully used to detect TZ in spiked food samples. Comparison of important indexes of this sensor with those of previous electrochemical sensors for TZ revealed that this sensor showed improved performance. This surface-imprinted sensor provides an ultrasensitive, highly specific, effective, and low-cost method for TZ determination in foodstuffs.

## 1. Introduction

**Figure 1:**
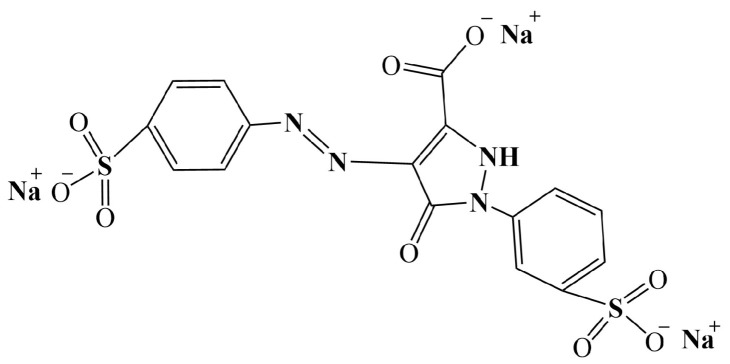
The molecular structure of tartrazine.

Tartrazine (TZ, Figure 1) is an artificially synthesized azo pigment that has been widely used as a colorant in drinks, foods, cosmetics, and pharmaceuticals to make them more attractive. However, TZ shows genotoxicity towards DNA, hemoglobin, and lymphocytes, and neurotoxicity towards memory, learning, thought, and other neurobehavior. TZ can cause symptoms such as diarrhea, allergies, eczema, migraines, dysphoria, and melancholia, and even cancer after long-term excessive ingestion. On account of these risks, the use of TZ is strictly regulated, and the maximum intake of TZ should not exceed 7.5 mg/kg daily [1,2]. It is very important to determine TZ levels in materials to ensure consumer safety. Recently, a diverse range of detection systems for TZ have been developed to overcome the drawbacks of common analytical techniques, such as low sensitivity and anti-interference ability, complex programming, and lengthy analysis time. Among the developed approaches, the electrochemical method exhibits obvious advantages in terms of sensitivity, accuracy, time consumption, convenience, economy, and miniaturization [3].

**Scheme 1:**
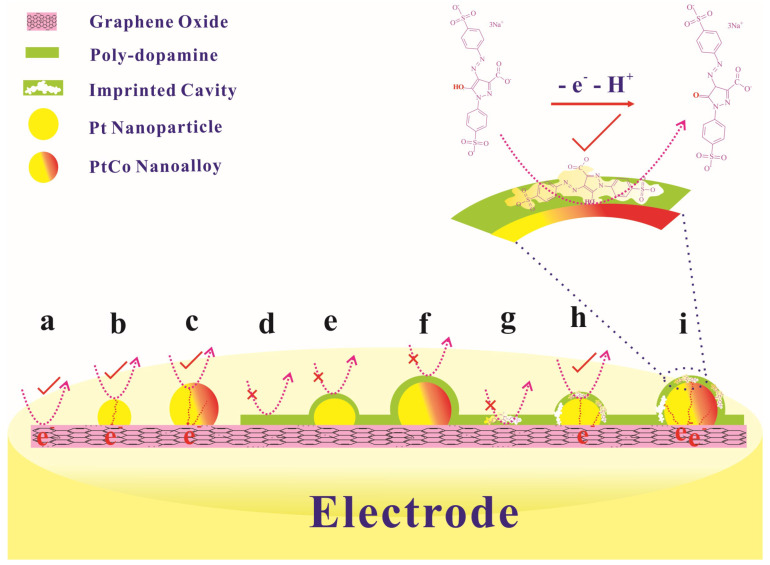
Schematic illustration of various electrochemical interfaces with TZ. (a) GO, (b) GO–Pt, (c) GO–PtCo, (d) GO@NIPDA, (e) GO–Pt@NIPDA, (f) GO–PtCo@NIPDA, (g) GO@MIPDA, (h) GO–Pt@MIPDA, and (i) GO–PtCo@MIPDA.

Sensitivity and specificity are two important factors governing the performance of electrochemical sensors [4]. TZ contains an electrochemically active phenolic hydroxyl group in its structure (Scheme 1) which can be directly used as the source of a detection signal at an electrode surface. However, the sensing performance depends on the properties of the electrode–TZ interface. Diverse surfaces have been constructed for TZ sensing (Table S1 in Supplementary Material). Generally, electrodes possessing surfaces without recognition elements have been studied which mainly exhibited anti-interference abilities based on the different electroactive potentials of interfering species. Four studies to date have used molecularly imprinted polymers (MIPs) as recognition elements in electrochemical sensors for TZ based on polypyrrole, MWNTs-IL@PtNPs, PmDB/PoPd, and acrylamide-CN, respectively (Table S1). The results of these studies indicated that electrodes with MIPs were easy to prepare, cost-effective, operated quickly and conveniently, and demonstrated higher stability and efficiency than other recognition techniques, including immunosensors and aptasensors. Thus, the use of MIPs combined with the development of materials and techniques to improve sensing abilities should attract increasing interest [5,6].

MIPs are also called “artificial antibodies” and have been used in solid-phase extraction, molecular recognition, chromatographic separation, drug delivery, biological engineering, and biomimetic sensing. For MIP-based electrochemical sensors, the electrocatalytic activity and microenvironment of the imprinted cavities are the most important factors influencing sensor performance [7,8]. Therefore, the matrix materials should be considered first. Two-dimensional graphene possesses outstanding electrical conductivity, and some its composites display high electrocatalytic activity, making them promising for application in MIP sensors [9,10]. Furthermore, surface-imprinted nanocomposites show remarkable advantages that improve the microenvironments of imprinted cavities compared with those of conventional imprinted hybrids, such as more complete template removal, higher binding capacities, faster binding kinetics, and more facile electron transfer [7,8,11].

A simple, rapid, and cost-effective method for quantitative analysis of TZ with high sensitivity and selectivity still needs to be developed. Herein, a novel MIP nanocomposite sensor for TZ is prepared by grafting an ultrathin membrane of molecularly imprinted polydopamine (MIPDA) onto the matrix surface of platinum cobalt nanoalloy-functionalized graphene oxide (GO–PtCo). The sensitivity and selectivity of the developed electrochemical sensor in TZ detection are investigated.

## 2. Materials and Methods

### 2.1. Reagents and Instruments

Tartrazine (TZ) and dopamine hydrochloride (DA) were purchased from Sigma-Aldrich (St. Louis, MO, USA). Graphite, hexachloroplatinatic acid hexahydrate (H_2_PtCl_6_·6H_2_O), cobalt chloride hexahydrate (CoCl_2_·6H_2_O), sodium borohydride (NaBH_4_), Allura Red, sunset yellow, amaranth, brilliant blue, indigo carmine, sodium salicylate, vitamin C, glucose, sucrose, citric acid, benzoic acid, phenol, and sodium acetate were purchased from the Aladdin Chemistry Co., Ltd. (Shanghai, China). Other chemicals were purchased from the Shanghai Chemical Reagent Co. (Shanghai, China). All chemicals were of analytical grade and used without further purification. The drink and food samples were purchased from local markets. Double-distilled deionized water was used throughout this work.

Electrochemical experiments were performed using a CHI 660D electrochemical workstation (Chenhua Instruments, Shanghai, China) with a conventional three-electrode system, including a modified GCE (diameter: 3 mm) as a working electrode, a Pt wire supporting electrode, and a saturated calomel reference electrode. Surface morphological images were recorded using a scanning electron microscope (S-4800, Hitachi, Japan) and a transmission electron microscope (JEM-3010, JEOL, Tokyo, Japan). All experiments were carried out at room temperature unless otherwise noted.

### 2.2. Fabrication of the Molecularly Imprinted Nanocomposites

Graphene oxide (GO) was prepared from graphite powder using a typical method with some modifications [12]. Typically, graphite (1.5 g), NaNO3 (1.5 g), and concentrated H2SO4 (69 mL) were stirred together in an ice bath. Next, KMnO4 (9 g) was slowly added to the mixture. The mixture was transferred to a water bath and stirred for 1 h at room temperature. Water (100 mL) was added slowly. The solution was heated to 90 °C and then held at this temperature for 30 min under stirring. Water (300 mL) was added, followed by the slow addition of H2O2 (10 mL, 30%). The yellow suspension was filtered, washed with 1 M HCl and water until the pH was 7.0, and then vacuum dried at 60 °C to obtain GO.

PtCo nanoalloy-decorated GO (GO–PtCo) was synthesized by consulting the reported method [13,14,15]. In brief, GO (12 mg) was dispersed in water (30 mL) by ultrasonication for 1 h. Then, H_2_PtCl_6_·6H_2_O (3.424 mg) and CoCl_2_·6H_2_O (0.523 mg) were added and the mixture was stirred for 24 h. Fresh NaBH_4_ (24 mg in 10 mL of water) was added quickly into the mixture, which was then stirred for 2 h. The products were collected by centrifugation, washed with deionized water to remove unreacted reagents and impurities, and then dispersed in water (20 mL). Other nanoalloy particles were prepared by adjusting the amounts of materials added to the precursor solution. GO–Pt and GO–Co were prepared by the same procedure in the absence of CoCl_2_·6H_2_O or H_2_PtCl_6_·6H_2_O, respectively.

In a typical synthesis of MIPDA-coated GO–PtCo (denoted GO–PtCo@MIPDA), DA (15 mg) and TZ (15 mg) (molar ratio of ~7.4:2.6) were dissolved in water (30 mL) under ultrasonication for 1 h in ice water. The mixture was added to the above GO–PtCo dispersion. The pH of the mixture was adjusted to 8.5 with Tris–HCl buffer solution. The mixture was strongly stirred for 24 h at room temperature. After the reaction, the product was centrifugally washed with water to remove any unreacted monomers, and then immersed in a basic mixture (*V*_ethanol_:*V*_concentrated ammonia_:*V*_water_ = 7:2:1) to extract the TZ template molecules. Washing was repeated until no template molecules were detected in the extraction solution, and then the product was rinsed thoroughly with distilled water. Other imprinted nanocomposites were synthesized via a similar process by changing the concentration of TZ template molecules or DA. For comparison, non-imprinted PDA-coated GO–PtCo was prepared by the same procedure in the absence of the TZ template molecules; the product was denoted as GO–PtCo@NIPDA. The template extraction process was also performed on the control GO–PtCo@NIPDA composite.

The GO@MIPDA, GO–Pt@MIPDA, GO@NIPDA, and GO–Pt@NIPDA nanocomposites were synthesized via a similar procedure to GO–PtCo@MIPDA and GO–PtCo@NIPDA using GO or GO–Pt as the matrix. Adsorption experiments were conducted as follows. Each material (3 mg) was added to TZ solution (4 mL; see Figure S7 for the concentrations used). After stirring for 20 min at room temperature, the mixture was centrifuged, the supernatant was collected, and its TZ content was determined by UV–Vis absorption spectroscopy. The adsorbed amount was calculated according to the formula *Q* = *V*(*c*_0_−*c*_S_)/*m*, where *V* represents the solution volume, *c*_0_ and *c*_S_ are the TZ concentrations before and after adsorption, respectively, and *m* is the mass of material.

### 2.3. Fabrication of Modified GCEs and Electrochemical Measurements

A bare GCE was polished with slurry alumina (0.3 μm and then 0.05 μm) and then washed with water with the aid of ultrasonication. GO–PtCo@MIPDA suspension (5 μL, 3 mg/mL in water) was carefully dropped onto the surface of the GCE. A GO–PtCo@MIPDA film-coated GCE (denoted GCE/GO–PtCo@MIPDA) was obtained through self-assembly during solvent evaporation in air. For repeated detection using one electrode, the electrode was dipped in agitated eluent solution (*V*_ethanol_:*V*_concentrated ammonia_:*V*_water_ = 7:2:1) for 12 min to recover the functional surface of GCE/GO–PtCo@MIPDA before the next measurement. Other modified electrodes, including GCE/GO, GCE/GO–Pt, GCE/GO–PtCo, GCE/GO@NIPDA, GCE/GO–Pt@NIPDA, GCE/GO–PtCo@NIPDA, GCE/GO@MIPDA, and GCE/GO–Pt@MIPDA, were prepared in a similar manner to GCE/GO–PtCo@MIPDA.

Each three-electrode system was assembled in a cell with 0.2 M acetate–sodium acetate (Hac–NaAc) buffer solution containing a certain concentration of TZ. After the sensor was immersed in solution for 10 min under mild magnetic stirring to reach adsorption equilibrium, a CV or DPV was recorded unless otherwise noted. For the detection of TZ in drink and food samples, corresponding 1.0 g/L solutions were used as detection samples. Before TZ detection in these samples, the pH of the prepared water samples was adjusted to 6.0, and the DPV peak corresponding to TZ in the real samples was recorded to reveal the amount of TZ in each sample.

## 3. Results

### 3.1. Characterization and Electrochemical Behavior of Nanocomposites

**Figure 2:**
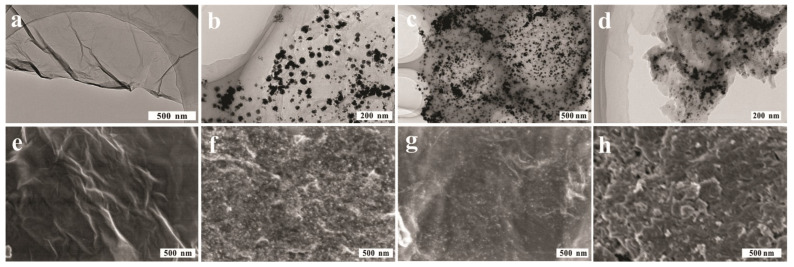
TEM and SEM images of (**a**,**e**) GO, (**b**,**f**) GO–PtCo, (**c**,**g**) GO–PtCo@MIPDA, and (**d**,**h**) GO–PtCo@NIPDA.

Graphene oxide (GO) is known as an ideal matrix for in situ nucleation, growth, and anchoring of naked metal nanoparticles because of its functional groups and lattice defects, ultrahigh mechanical strength, and large planar structure. A one-step method that involved reducing a metal precursor in GO solution was used to form the GO–PtCo composite in situ [13,14,15]. Spherical PtCo nanoalloy particles with a diameter of less than 25 nm were synthesized in situ on the GO surface (Figure 2b,f). The dispersion and size of nanoalloy particles are not very uniform and may be influenced by the surface groups and defects of GO which affect the microenvironment for lattice growth. However, we wanted to fabricate a bare atomic interface without any mediating ligands because this should have been beneficial for electrocatalysis. In the absence of cobalt chloride hexahydrate but otherwise identical conditions, tiny Pt nanoparticles with an almost uniform size of approximately 5 nm were distributed on the GO surface (Figure S1d). GO supporting pure Co nanoparticles was not produced in the absence of hexachloroplatinatic acid hexahydrate, which is consistent with previous results [13,14,15]. After monomeric self-polymerization, a melanin-like polydopamine with a cross-linked structure was coated on the GO surface [16,17,18,19]. Electron micrographs of the surfaces coated with MIPDA showed uniform contrast, revealing that the surfaces were fairly smooth and that the coating process was well-controlled (Figure 2c,g and Figure S1b,e). After coating with non-imprinted polydopamine (NIPDA), the surfaces exhibited partial agglomeration, indicating that sunset yellow (SY) affected the self-polymerization of dopamine hydrochloride (DA) on the GO surface to some extent (Figure 2d,h, and Figure S1c,f).

**Figure 3:**
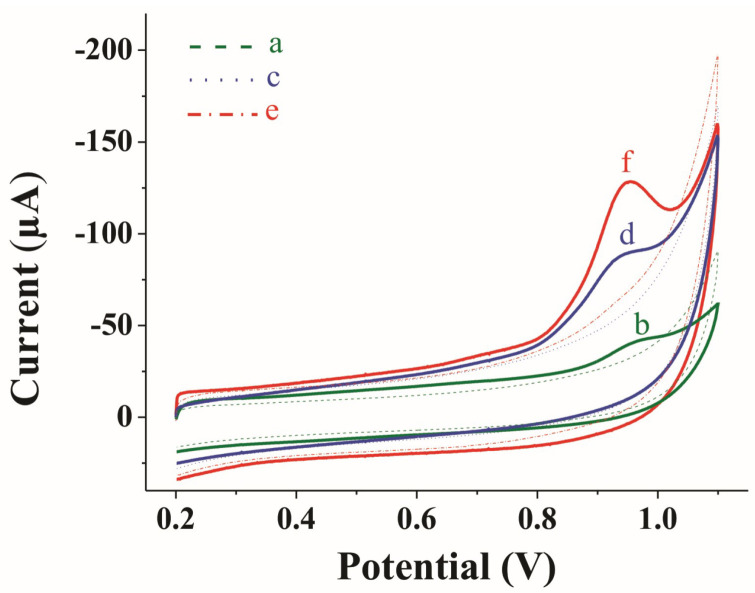
CVs of (a, b) GCE/GO, (c, d) GCE/GO–Pt, and (e, f) GCE/GO–PtCo with (a, c, e) 0 μM and (b, d, f) 9.00 μM TZ in acetate buffer solution (pH = 6.0). Scan rate: 0.1 V/s.

Cyclic voltammetry (CV) was employed to investigate the electrochemical performance of the materials (Figure 3). GO exhibited a weak anodic response to TZ at +0.962 V, revealing an irreversible electrochemical process (Scheme 1). After decoration with Pt nanoparticles, the peak strength and overpotential slightly increased and decreased, respectively. Interestingly, the peak strength was further raised by 2.809 times after PtCo decoration, indicating that the nanocomposite displayed prominent electrocatalytic activity towards TZ. This property might be related to the bimetallic surface with good heterogeneous hybridization allowing efficient charge transfer [15], potentially offering high sensitivity. However, interference signals from coexisting substances might accompany such increases, especially those with similar response potentials. Therefore, it is important to find efficient and simple approaches to produce surfaces with specific recognition abilities.

**Figure 4:**
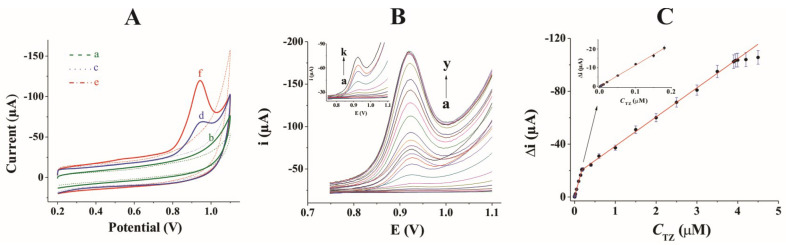
(**A**) CVs of (a) GCE/GO@NIPDA, (b) GCE/GO@MIPDA, (c) GCE/GO–Pt@NIPDA, (d) GCE/GO–Pt@MIPDA, (e) GCE/GO–PtCo@NIPDA, and (f) GCE/GO–PtCo@MIPDA in the presence of 9.00 μM TZ. (**B**) DPVs of GCE/GO–PtCo@MIPDA measured at TZ concentrations of (a–y): 0, 0.001, 0.002, 0.003, 0.005, 0.01, 0.02, 0.05, 0.1, 0.15, 0.18, 0.2, 0.4, 0.6, 1.0, 1.5, 2.0, 2.5, 3.0, 3.5, 3.9, 3.95, 4.0, 4.2, and 4.5 μM. (**C**) Calibration curves of peak current versus TZ concentration for GCE/GO–PtCo@MIPDA. Electrolyte: 0.2 M (pH = 6.0) acetate buffer solution. Scan rate: 0.1 V/s. Pulse width: 0.2 s. Pulse period: 0.5 s. Amplitude: 0.05 V.

Surface-imprinted polymers are increasingly being used as specific recognition elements. Considering the attractive properties of PDA, including abundant functional groups, a cross-linked structure, and robust adhesiveness [16,17,18,19], it is expected that surface-imprinted materials with good performance could be obtained using PDA coatings. The electrochemical responses of various surface-coated composites to TZ are presented in Figure 4A. The original responses of GO, GO–Pt, and GO–PtCo to TZ disappeared after coating with NIPDA. This indicates that the NIPDA layer had no electrocatalytic activity towards TZ and instead exhibited a marked electronic blocking effect between the inner matrix and outer TZ. Therefore, NIPDA-coated nanocomposites are unsuitable for TZ sensing applications. Conversely, peak strengths of GCE/GO–Pt and GCE/GO–PtCo (GCE is the underlying glassy carbon electrode coated with the composites) were enhanced by 1.235 and 1.523 times, respectively, after MIPDA coating (Figure 2 and Figure S1). These results imply that the vacant cavities could anchor a large amount of configuration-suitable TZ, whose electroactive phenolic hydroxyl group could be efficiently oxidized via the Pt and PtCo surfaces and whose electrons could be readily transferred to the electrode (Scheme 1). It should be pointed out that the original response peak of GCE/GO to TZ disappeared after MIPDA coating. This might be because the electroactive sites were enveloped during DA self-polymerization and the imprinted cavities were unsuitable for electron transfer from TZ molecules. According to the CV analysis, GCE/GO–PtCo@MIPDA was selected as a TZ sensor because it displayed the greatest response to TZ.

### 3.2. Optimization of Conditions

According to the above investigation, it is clear that GCE/GO–PtCo@MIPDA had a high sensing ability for TZ. To achieve better sensing performance, the experimental conditions should be optimized, including the nanoalloy composition, the molar ratio of DA to template molecules, the amount of DA monomer, incubation time, elution time, pH, and adsorption kinetics.

#### 3.2.1. Optimization of the Nanocomposite

The GO skeleton is composed of sp^2^-bonded carbon atoms arranged in a two-dimensional honeycomb lattice that is one atom thick and has numerous oxygen-containing groups on the basal plane and edges [12]. It is well known that the electrocatalytic performance of GO-supported nanoalloys is closely related to their composition [15]. To investigate this effect, the molar ratio of Pt to Co in the precursor solution was altered while keeping the total molar amount constant. The corresponding current for TZ was recorded. It was observed that the amount of surface PtCo decreased with an increasing amount of Co in the precursor solution, indicating that Co played an important role in constructing the bimetallic nanocomposite (Figure S2A,B). In addition, Co NPs did not form on the GO surface under the same conditions. With increasing amounts of Co in the bimetallic alloy, the current strength for TZ first increased and then decreased (Figure S2C). The maximum current was obtained using a precursor molar ratio of 3:1, indicating that the presence of Co was a vital factor influencing the electrocatalytic performance of the sensor.

MIPDA was prepared via DA self-polymerization. The content of imprinted cavities in the MIP was directly affected by the ratio of template molecules to functional monomers, which further affects the rebinding and recognition abilities of the prepared sensor. Therefore, the currents of imprinted nanocomposites produced using a series of DA:TZ molar ratios of 9:1, 8.5:1.5, 8:2, 7.4:2.6, 7:3, 6.5:3.5, and 6:4 in MIPDA synthesis were recorded (Figure S3A); the total molar amount was fixed at 0.1072 mmol. The obtained imprinted nanocomposites exhibited a gradual decrease in TZ signal as the DA:TZ molar ratio increased from 7.4:2.6 to 9:1. The reason for this might be that the MIPDA contained less specific recognition cavities and excess PDA hindered electron transfer. When the molar ratio was lower than 7.4:2.6, the current response decreased obviously, possibly because excess template molecules could not combine with the DA and hindered the polymerization, resulting in a lower amount of effective binding cavities. Therefore, the optimal DA:TZ ratio was 7.4:2.6 (15 mg of TZ and 15 mg of DA in the precursor solution), which was used in subsequent experiments.

The peak currents obtained when the quantity of DA was altered while keeping the DA:TZ ratio at 7.4:2.6 during the self-polymerization process for 24 h are shown in Figure S3B. The current response increased with DA quantity from 5 to 15 mg and then decreased as the DA amount increased further. The maximum current response was obtained for a DA quantity of 15 mg, which corresponded to the typical MIP nanocomposite. Therefore, the specific amounts of DA and TZ used in the composite were both 15 mg.

It is known that the thickness of a PDA film can be controlled by modulating the polymerization time, which also affects the depth of the imprinted cavities. Therefore, the synthesis period of the MIP nanocomposite was studied. As shown in Figure S3C, the maximum current response was clearly achieved with the MIPDA nanocomposite prepared over 24 h. When the reaction period was longer than 24 h, the current response decreased, suggesting that the layer was too thick to remove the template molecules completely and readily transfer electrons. Therefore, a reaction time of 24 h was used to produce an imprinted membrane of suitable thickness. Meanwhile, the NIP nanocomposite showed a dramatic decrease in TZ signal with increasing DA quantity after exceeding the amount of 0.5 mg (Figure S3D). This result further indicates that the thickness of the MIP is an important parameter affecting the sensitivity and stability of the constructed sensor.

#### 3.2.2. Incubation and Elution Times of the MIPDA Sensor

The GCE/GO–PtCo@MIPDA sensor was incubated in TZ solution for different times at room temperature and then the response current was measured. As shown in Figure S4A, the current signal gradually increased with lengthening incubation time and achieved its maximum value when the incubation time was 10 min. This suggests that the adsorption equilibrium can be reached within 10 min, meaning that the prepared sensor possesses fast rebinding dynamics. Thus, to achieve high sensitivity and save assaying time, an incubation time of 10 min was chosen.

To reuse GCE/GO–PtCo@MIPDA, the adsorbed TZ needed to be removed to recover its molecular recognition ability. In this experiment, a basic mixture (*V*_ethanol_:*V*_concentrated ammonia_:*V*_water_ = 7:2:1) was used as the eluent to remove TZ; good results were achieved. Figure S4B demonstrates the relationship between the extraction time and effective sensor recovery. When the extraction time was longer than 12 min, the peak current originating from adsorbed TZ almost disappeared. Therefore, 12 min was chosen as the optimal extraction time to ensure complete elution of TZ so that the imprinted cavities could show excellent recognition ability and a maximum current response to TZ in solution upon reuse.

#### 3.2.3. Solution pH

The pH of the buffer solution was also optimized for quantitative determination of TZ (Figure S5). The response currents in the pH range of 5.0–7.5 using acetate buffer solutions indicated that the anode peak current (*i*_pa_) increased with pH from 5.0 to 6.0 and reached a maximum at pH = 6.0. Then, *i*_pa_ decreased as the pH increased from 6.0 to 7.5 (Figure S5B). Thereby, a pH of 6.0 was chosen as the optimal value for the quantitative determination of TZ. Furthermore, as the pH of the medium was gradually increased, the oxidation peak potential of TZ shifted to a more negative voltage, suggesting that protons were involved in the electrochemical reaction. Over the pH range of 5.0–7.5, the peak potential of TZ at GCE/GO–PtCo@MIPDA shifted linearly in the negative direction with increasing pH, with a slope of –44.5 mV/pH (Figure S5C), indicating that one proton is directly involved in the oxidation of TZ (Scheme 1). The deviation of this slope from the theoretical value could be ascribed to the influence of PDA and the slowing of the electrode reaction.

#### 3.2.4. Adsorption Characteristics

Adsorption experiments were used to evaluate the molecular imprinting effect of TZ. The adsorption kinetics of TZ on GCE/GO–PtCo@MIPDA were investigated at different time intervals (Figure S6A). The adsorbed amount (Q) of TZ on GO–PtCo@MIPDA increased rapidly during the first 12 min, confirming its fast adsorption kinetics and the adsorption process almost reached equilibrium in this time. In contrast, GO–PtCo@NIPDA exhibited increasing Q during the first 8 min, indicating the occurrence of non-specific adsorption. In contrast, GO–PtCo had no obvious influence on the Q of TZ against adsorption time. This is because GO–PtCo had no imprinted cavities and showed low resistance to non-specific binding.

The adsorption isotherms of TZ on GO–PtCo@MIPDA, GO–PtCo@NIPDA, and GO-PtCo were measured at different initial concentrations of TZ to investigate their binding capacities. As shown in Figure S6B, the amount of TZ adsorbed on GO–PtCo@MIPDA increased with TZ concentration from 0.015 to 0.175 mM and reached saturated adsorption over 0.175 mM. In this case, the experimental maximum adsorption capacity was 80.1 μmol/g. For GO–PtCo@NIPDA and GO–PtCo, the amount of TZ adsorbed changed more slowly with increasing TZ concentration. Similarly, when the TZ concentration exceeded 0.08 and 0.015 mM for GO–PtCo@NIPDA and GO–PtCo, respectively, the adsorption curves exhibited a plateau, indicating that saturated adsorption was achieved. The maximum adsorption capacities of GO–PtCo@NIPDA and GO–PtCo were 5.9 and 0.35 μmol/g, respectively, which were much lower than that of the MIP, suggesting lower binding affinity for TZ. These results could be explained by the fact that the imprinting process of GO–PtCo@MIPDA formed specific recognition cavities that showed a memory function and high adsorption capacity for TZ. In contrast, for GO–PtCo@NIPDA and GO–PtCo, non-specific adsorption was dominant because of their lack of TZ recognition sites. Therefore, the amount of TZ adsorbed by these composites was low.

### 3.3. Analytical Performance

#### 3.3.1. Calibration Curve

Differential pulse voltammetry (DPV) gives better peak shape and higher sensitivity than CV. As shown in Figure 4B, under optimum conditions, the change of peak current (i_p_) from GCE/GO–PtCo@MIPDA exhibited linear relationships with TZ concentration (C) in the ranges of 0.003–0.180 and 0.180–3.950 µM, with regression equations of i_p_ (μA) = −113.3156 × C (μM) − 0.03219 (R^2^ = 0.9983) and i_p_ (μA) = −22.0380 × C (μM) − 16.4604 (R^2^ = 0.9989), respectively. The detection limit was 1.1 nM (S/N = 3). When C was above 3.950 μM, the calibration curve gradually deviated from the straight line, indicating that saturated adsorption was gradually reached. A linear range of 0.040–9.650 μM and detection limit of 16.5 nM were obtained for GCE/GO–PtCo (Figure S7A). The detection limit of GCE/GO–PtCo@MIPDA was 14.8 times lower than that of GCE/GO–PtCo, demonstrating the considerable improvement induced by the MIPDA layer. GCE/GO–PtCo@NIPDA did not display a current peak or linear response to TZ, indicating that the NIPDA overlayer acted as an electronic barrier with no effective binding sites for TZ.

#### 3.3.2. Sensor Selectivity, Reproducibility, and Stability

**Figure 5:**
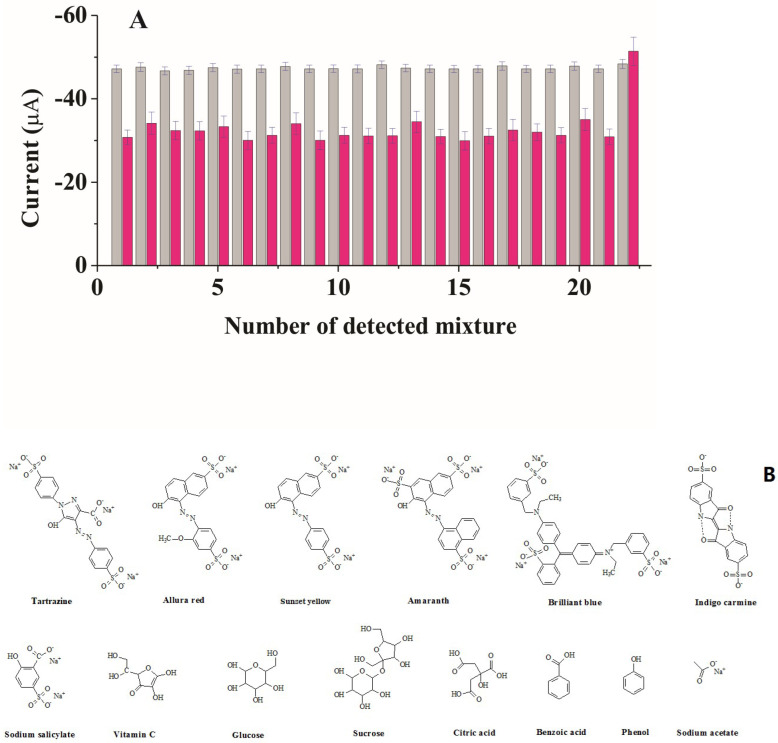
(**A**) Influence of coexisting substances on the electrochemical response of GCE/GO–PtCo@MIPDA (grey) and GCE/GO–PtCo (pink) to TZ. Solution composition: (1) 3.5 µM TZ in 0.2 M acetate buffer solution (pH = 6.0); (2 to 21) with 0.1 mM of (2) Allura Red, (3) sunset yellow, (4) amaranth, (5) brilliant blue, (6) indigo carmine, (7) sodium salicylate, (8) vitamin C, (9) glucose, (10) sucrose, (11) citric acid, (12) benzoic acid, (13) phenol, (14) sodium acetate, (15) Ca^2+^, (16) K^+^, (17) Fe^3+^, (18) Zn^2+^, (19) SO_4_^2−^, (20) NO_2_^−^, (21) Cl^−^, and (22) a mixture of (1) and the other 20 kinds of interferents (50 µM each). (**B**) Molecular structures of some of the analogs.

The binding selectivity of GCE/GO–PtCo@MIPDA for TZ and other structural analogs, which are possible coexisting additives that may be present with higher concentrations, was evaluated by competitive experiments. As shown in Figure 5A, the peak current after incubation in TZ mixtures with interfering substances showed no obvious discrepancy with that after incubation in pure TZ solution, implying that the MIPDA sensor displayed a highly specific recognition ability for TZ and that it was not affected by interfering species, even structural analogs. In comparison, some coexisting species seriously disturbed the current response of GO–PtCo to TZ (Figure 5A), which was caused by its lack of a specific recognition element. The highly selective electrochemical surface of GCE/GO–PtCo@MIPDA should originate from the synergistic effect of the MIPDA cavities that matched TZ and rest of the MIPDA layer acting as an electronic barrier to outside molecules. These results also suggest that the binding cavities in the imprinted surface were only complementary with the size and shape of TZ, which might facilitate the electroactive sensor response via covalent or non-covalent interactions between TZ and cross-linked PDA.

The reproducibility of the response of GCE/GO–PtCo@MIPDA was estimated by detecting a 0.15 µM TZ solution with six different electrodes prepared under the same conditions. The current response showed a relative standard deviation (RSD) of 3.25% for six independent measurements. A RSD of 4.17% was obtained after 20 cycles of washing and measurement, and the signal strength decreased to 95.00% of the initial strength after 75 cycles, indicating the good reproducibility and excellent recyclability of the sensor. Moreover, the stability of the sensor was evaluated by storing six independently fabricated electrodes for six weeks at room temperature. The peak current did not change markedly over the first two weeks. After six weeks, the average signal was 96.04% of the initial response. These measurements indicate that GCE/GO–PtCo@MIPDA possesses suitable reproducibility and stability for successful use as a sensor.

#### 3.3.3. Application

The practical application of the electrochemical sensor was assessed by detecting the TZ levels of different samples (Table 1). The recoveries of six independent experiments ranged from 95.3% to 105.1%, and the RSD was below 4.37%. These results reveal the good accuracy and reliability of the sensor and its ready ability to detect TZ in real foodstuffs. The detection limit of the sensor of 1.1 nM is sufficient to detect TZ in real samples, which are allowed to contain from 50 to 500 μg/g [2]. Overall, GCE/GO–PtCo@MIPDA displays excellent electrochemical performance and is suitable for practical application in TZ detection.

### 3.4. Comparison of Sensor Performance

Over the past decade, TZ has been detected using numerous electrochemical methods. The analytical performance of the MIPDA sensor for TZ was compared with that of previous TZ assays using well-known technical indicators for analytical methods, including linear interval, detection limit, recognition component, anti-interference ability, and recovery from real samples (Table S1). The MIPDA sensor is more sensitive than most others previously reported. Ultrasensitive TZ detection was achieved because of the facile electron transfer between the TZ-enriching cavities that readily captured TZ in a suitable configuration and the highly electroactive surface of GO–PtCo. In addition, the anti-interference ability of our sensor was the best achieved to date. The selective electrochemical sensor for TZ was achieved through the synergistic effects of the MIPDA cavities complementary to TZ and the anti-interference screening ability of the rest of the MIPDA coating. The sensor also exhibited improved performance in terms of linear interval and recovery compared with those of previously reported electrochemical sensors for TZ. This comparison confirmed that our GO–PtCo@MIPDA composite is an appropriate platform for electrochemical sensing of TZ. The prepared MIPDA sensor displayed a wide linear range with a low detection limit, indicating its promise for quantitative assaying of TZ. In addition, the GO–PtCo@MIPDA composite has the advantages of low production cost and a facile one-pot preparation procedure in aqueous solution at ambient temperature, which is environmentally friendly.

### 3.5. Discussion of Electrochemical Sensing Surfaces

The surface of the GO-modified electrode exhibited a small anodic peak response to TZ, which was not displayed by the bare electrode surface, indicating that there are electrochemically active sites on GO for configuration-suitable molecules within the diffusion layer (Figure 3, curve b, and Scheme 1a). Larger anodic peaks were obtained on the corresponding surfaces of Pt- or PtCo-decorated GO (Figure 3, curves d and f, respectively, and Scheme 1b,c, respectively). These results clearly show that Pt and PtCo provided more active sites for TZ than GO, supporting the idea that incorporating metal nanoparticles is an effective strategy for increasing the electrocatalytic activity of GO [20,21,22,23]. In addition, the presence of Co in the nanoalloy further influenced the electrocatalytic activity of the sensor, which is a result worthy of further research.

Under alkaline conditions (pH > 7.5), DA is first oxidized by oxygen to DA quinone, followed by 1,4-Michael-type intramolecular cyclization to yield leucodopaminechrome. Leucodopaminechrome undergoes further oxidization and intramolecular rearrangement to form 5,6-dihydroxyindole and its isomer, which subsequently polymerize to give PDA, a melanin-like polymer with a cross-linked structure. PDA obtained by self-polymerization of DA tends to coat diverse substrates as a thin film, especially GO and metallic surfaces. It is generally accepted that the catechol group plays the central role in the mussel-mimicking behavior of PDA [16,17]. The current peaks disappeared after coating NIPDA on the surface of GO, GO–Pt, and GO–PtCo (Figure 4, curves a, c, and e, respectively, and Scheme 1d–f, respectively), meaning that NIPDA showed no electrocatalytic activity towards TZ and prevented the access of TZ to the conductive matrix. After surface coating of GO–Pt and GO–PtCo with MIPDA, the peak strengths for TZ were obviously enhanced over those of GCE/GO-Pt and GCE/GO-PtCo, respectively, which was attributed to the imprinted cavities in MIPDA (Figure 4, curves d and f, respectively; Figure 3, curves d and f, respectively; and Scheme 1h,i, respectively). For GO@MIPDA, the original peak response of GO to TZ disappeared, indicating that the original electroactive sites were used as grafting sites and covered by electrochemically inert PDA (Figure 4, curve b; Figure 3, curve b; and Scheme 1g). Overall, GO–PtCo@MIPDA showed the greatest response to TZ, indicating that it displays the most promise for application as a TZ sensor.

Usually, surface MIP composites are prepared by polymerizing cross-linkers, functional monomers, and template molecules through covalent or non-covalent interactions via various routes, including free-radical polymerization, reversible addition–fragmentation chain transfer polymerization, atom transfer radical polymerization, and the sol–gel method. An imprinted hybrid material with recognition ability is obtained after removal of the embedded template molecules [7]. Here, the surface MIPDA was fabricated in one step through DA self-polymerization in the presence of template molecules, which avoided the tedious multiple preparation steps needed to produce previous GO-based MIPs, representing an improved fabrication technology compared to those reported previously [8,11]. The whole synthesis was carried out in aqueous solution at ambient temperature under mild reaction conditions that were environmentally friendly. Further development of PDA is warranted because of its extensive adherence ability and promising behavior as a new functionalized surface.

To construct imprinted composites with effective electron transfer surfaces, good interfacial bonding and interactions between the matrix and MIPs are very important. TZ was first mixed with DA in water to induce molecular self-assembly via intermolecular forces, such as hydrogen-bonding and π–π and electrostatic interactions. During DA self-polymerization, template TZ molecules were embedded in the PDA through intermolecular forces. The embedded TZ molecules were closely anchored to the GO–PtCo surface with a configuration suitable to allow effective electron transfer. Due to the small dimensions and the extremely high surface-to-volume ratio of the imprinted molecules, most were situated at or close to the matrix or metal surface to form a stable state. After the removal of embedded template molecules, three-dimensional imprinted cavities complementary to the template molecules in terms of shape, size, and functional groups were formed; these cavities could rebind template molecules with high specificity. This laid the foundation for the manifested high selectivity of the electrochemical sensor, combined with the electrocatalytically inert MIPDA surface, which helped to prevent interference from other materials. Therefore, GO–PtCo@MIPDA displayed an obvious preconcentration effect by binding to TZ in a suitable configuration, increasing the local amount of TZ compared with that in the diffusion layer near GO–PtCo. The electrochemical behavior of GO–PtCo@MIPDA towards TZ indicated that the layer of MIPDA must be ultrathin and that the PtCo surface in the cavities might be nearly bare, which increased the electrocatalytic activity for TZ. Therefore, the strongest electrical sensing signal was effectively transferred to the electrode via the highly specific GO–PtCo@MIPDA interface. These interesting phenomena could be ascribed to the combined effects of the highly electroactive surface of PtCo and the specificity of the imprinted cavities for TZ [24,25,26].

Overall, GO–PtCo@MIPDA combined the synergistic effects of the conductive matrix of GO, high electroactivity of PtCo, specific recognition cavities in MIPDA, and the blocking effect of MIPDA to interfering molecules. These combined features led to the achieved excellent electrochemical response and selectivity of GO–PtCo@MIPDA, making it attractive for sensing applications.

## 4. Conclusions

PtCo nanoalloy particles were synthesized on the surface of GO in situ and the obtained nanocomposite exhibited facile electrocatalysis of TZ. After the surface of GO–PtCo was coated with MIPDA, an electrochemical sensor for TZ with ultra-high sensitivity, high selectivity, good reproducibility, and long-term stability was obtained. The ultra-high sensitivity was ascribed to the facile electron transfer between the TZ-enriched cavities with a suitable configuration and the highly electrocatalytically active surface of GO–PtCo. The highly selective electrochemical surface for TZ was achieved through the MIPDA cavities that specifically matched TZ and the rest of the PDA coating behaving as an electronic barrier to other molecules. The good reproducibility and stability obtained were also related to the synergistic effect between the GO matrix and the imprinted polymer. This sensor showed potential for use in TZ determination and represents a strategy that may be adopted for other molecules. In the future, it will be important to research further heterogeneous composites of nanoalloys and graphene, the imprinting applications of polydopamine, signal transduction, and synergistic sensors.

## Figures and Tables

**Table 1:** Detection results for TZ in samples using GCE/GO–PtCo@MIPDA (*n* = 6).

Samples	TZ Added (µM)	TZ Expected (µM)	TZ Found (µM)	Recovery (%)	RSD (%)
Orangeade	0	-	0.061	-	-
	0.090	0.151	0.147	97.4	3.52
	0.500	0.561	0.550	98.0	2.83
Yellow wine	0	-	0.027	-	-
	0.090	0.117	0.119	101.7	1.65
	0.500	0.527	0.533	101.1	2.37
Ice cream	0	-	0.020	-	-
	0.090	0.110	0.106	96.4	4.08
	0.500	0.520	0.507	97.5	3.62
Jelly	0	-	0.075	-	-
	0.090	0.165	0.172	104.2	4.37
	0.500	0.575	0.568	98.8	3.55
Instant juice powder	0	-	0.047	-	-
	0.090	0.137	0.144	105.1	4.30
	0.500	0.547	0.549	100.4	1.18
Candy	0	-	0.038	-	-
	0.090	0.128	0.124	96.9	3.77
	0.500	0.538	0.519	96.5	3.91
Cookie	0	-	0.058	-	-
	0.090	0.148	0.141	95.3	4.36
	0.500	0.558	0.535	95.9	4.09

Average of six detection results obtained using the developed sensor. RSD = relative standard deviation.

## Data Availability

Raw data presented in this study are available on request from the corresponding author.

## Supplementary Materials

The following supporting information can be downloaded at: https://www.mdpi.com/article/10.3390/bios12050326/s1, Figure S1: TEM images of (a) GO, (b) GO@MIPDA, (c) GO@NIPDA, (d) GO–Pt, (e) GO–Pt@MIPDA, and (f) GO–Pt@NIPDA; Figure S2: TEM images of GO–PtCo produced using different molar ratios of H_2_PtCl_6_·6H_2_O to CoCl_2_·6H_2_O of (A) 1:1 and (B) 1:3 in precursor solution. (C) The relationship between the molar ratio of H_2_PtCl_6_·6H_2_O to CoCl_2_·6H_2_O in precursor solution and the current strength for TZ (9.00 μM); Figure S3: Effects of different (A) molar ratios of template molecules to functional monomers, (B) weights of DA in precursor solutions, and (C) synthesis periods on the current response of the MIPDA–nanocomposite-modified electrodes to 3.50 μM of TZ. (D) Effect of the weight of DA added to the precursor solution on the current response of the NIPDA–nanocomposite-modified electrode to 3.50 μM of TZ. Error bars are standard deviations across three repeated experiments; Figure S4: Effects of the (A) incubation time and (B) elution time on the response current from GCE/GO–PtCo@MIPDA. The concentration of TZ was 3.50 μM. Error bars are standard deviations across three repeated experiments; Figure S5: (A) CVs of GCE/GO–PtCo@MIPDA in 0.2 M acetate buffer solutions with 3.50 μM TZ and pHs of (a) 5.0, (b) 5.5, (c) 6.0, (d) 6.5, (e) 7.0, and (f) 7.5. (B) Relationship between the peak current (*i*_pa_) of TZ and pH. (C) Relationship between the peak potential of TZ and pH. Scan rate: 0.1 V/s; Figure S6: (A) Adsorption kinetics in 0.20 mM TZ solution and (B) adsorption isotherms of various composite materials; Figure S7: (A) DPVs for GCE/GO–PtCo exposed to TZ concentrations of (from a–j): 0, 0.01, 0.02, 0.03, 0.04, 0.05, 0.07, 0.1, 0.2, 0.5, 1.0, 2.0, 4.0, 7.0, 9.5, 9.65, 9.7, 10.5, and 13.0 μM. (B) Calibration curves of peak current versus TZ concentrations at GCE/GO–PtCo. Electrolyte solution: 0.2 M HAc–NaAc (pH = 6.0). Pulse width: 0.2 s. Pulse period: 0.5 s. Amplitude: 0.05 V; Table S1: Comparison of the analytical performance between the proposed method and graphene/MIP-based electrochemistry methods used in the past decade [27,28,29,30,31,32,33,34,35,36,37,38,39,40,41,42,43,44,45,46,47,48,49,50,51,52,53].
