# Supplementary material for: Specific and Sensitive Detection of Tartrazine on the Electrochemical Interface of a Molecularly Imprinted Polydopamine-Coated PtCo Nanoalloy on Graphene Oxide
Source: Biosensors (Basel). 2022 May 11;12(5):326. doi: 10.3390/bios12050326 (PMC9138349; doi:10.3390/bios12050326)
Supplement: Supplementary file 1 [file biosensors-12-00326-s001.zip › biosensors-1708557-supplementary.pdf]

Supplementary Materials

Title: Specific and sensitive detection of tartrazine on the electrochemical interface of a molecularly imprinted polydopamine-coated PtCo nanoalloy on graphene oxide

Shuwen Cheng [1,†], Danyao Tang [1,†], Yi Zhang [1,4,†], Libin Xu [1], Kunping Liu[3], Ke-Jing Huang [2,*], and Zheng-Zhi Yin [1,*]

[1] *College of Biological, Chemical Sciences and Engineering, Jiaxing University, Jiaxing 314001, Zhejiang Province, China*

[2] *Key Laboratory of Chemistry and Engineering of Forest Products, Guangxi Key Laboratory of Chemistry and Engineering of Forest Products, Key Laboratory of Guangxi Colleges and Universities for Food Safety and Pharmaceutical Analytical Chemistry, School of Chemistry and Chemical and Engineering, Guangxi University for Nationalities, Nanning 530008, China*

[3] *Key Laboratory of Medicinal and Edible Plants Resources Development of Sichuan Education Department, Sichuan Industrial Institute of Antibiotics, Chengdu University, Chengdu, 610106, China*

[4] *College of Chemical Engineering, Zhejiang University of Technology, Hangzhou 310014, China*

[*] Corresponding author.
E-mail address: yinzhengzhi@zjxu.edu.cn (Z.-Z. Yin). kejinghuang@163.com (K.-J. Huang).

[†] These authors are contributed equally to this work.

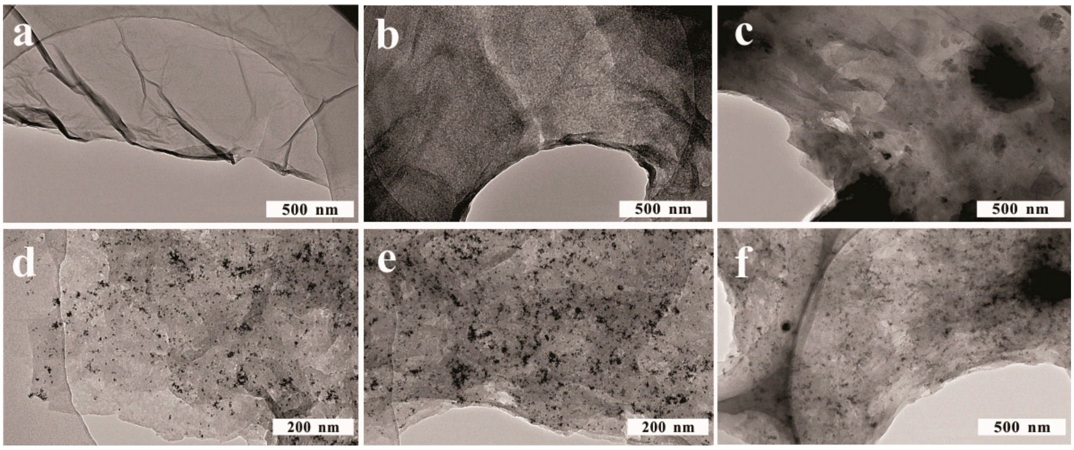

Figure S1. TEM images of (a) GO, (b) GO@MIPDA, (c) GO@NIPDA, (d) GO–Pt, (e) GO–Pt@MIPDA, and (f) GO–Pt@NIPDA.

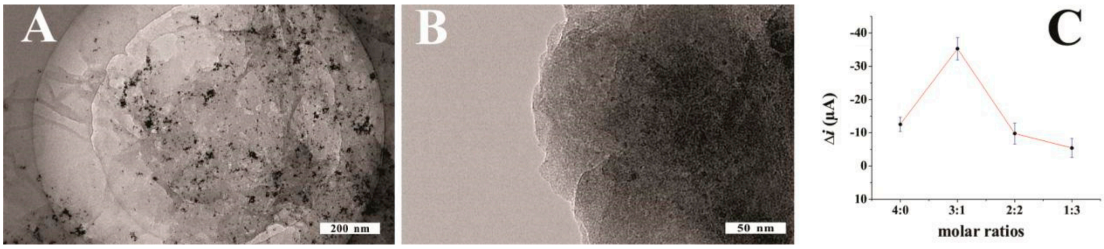

Figure S2. TEM images of GO–PtCo produced using different molar ratios of $H_2PtCl_6·6H_2O$ to $CoCl_2·6H_2O$ of (A) 1:1 and (B) 1:3 in precursor solution. (C) The relationship between the molar ratio of $H_2PtCl_6·6H_2O$ to $CoCl_2·6H_2O$ in precursor solution and the current strength for TZ (9.00 μM).

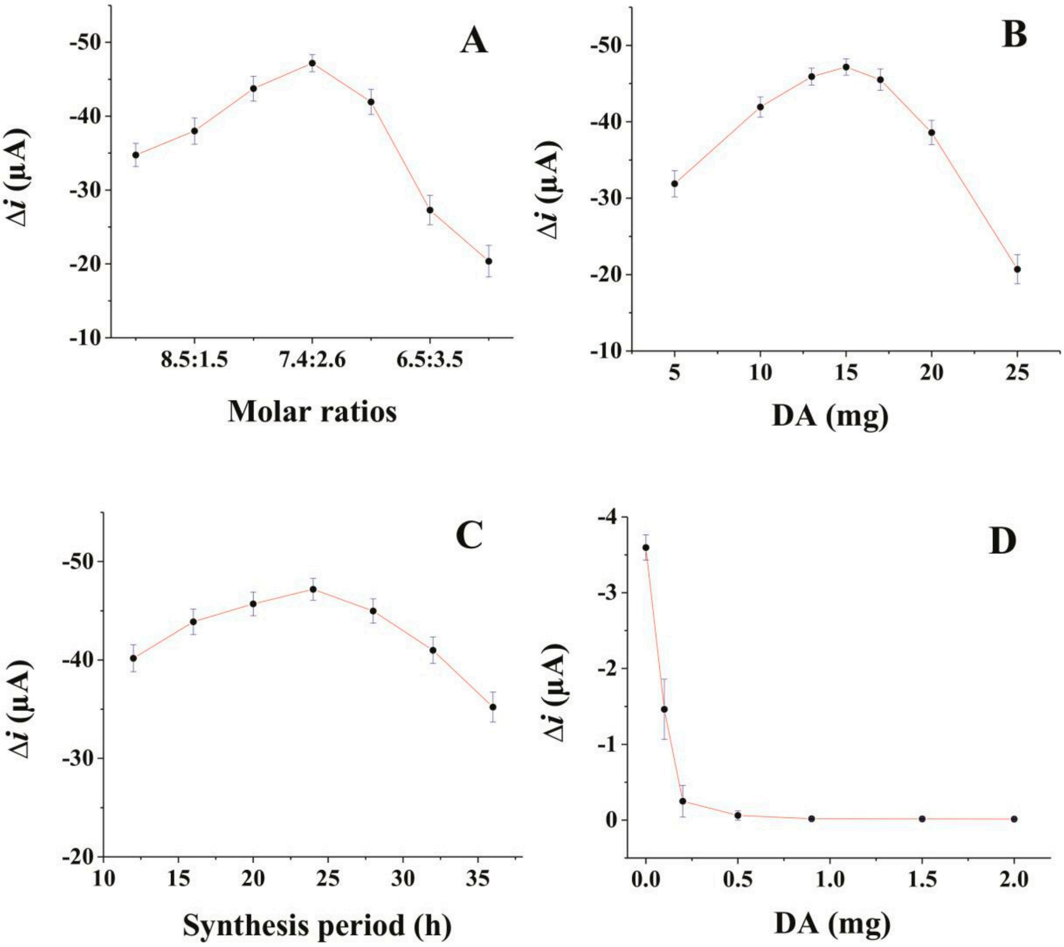

Figure S3. Effects of different (A) molar ratios of template molecules to functional monomers, (B) weights of DA in precursor solution, and (C) synthesis periods on the current response of the MIPDA–nanocomposite-modified electrodes to 3.50 μM of TZ. (D) Effect of the weight of DA added to the precursor solution on the current response of the NIPDA–nanocomposite-modified electrode to 3.50 μM of TZ. Error bars are standard deviations across three repeated experiments.

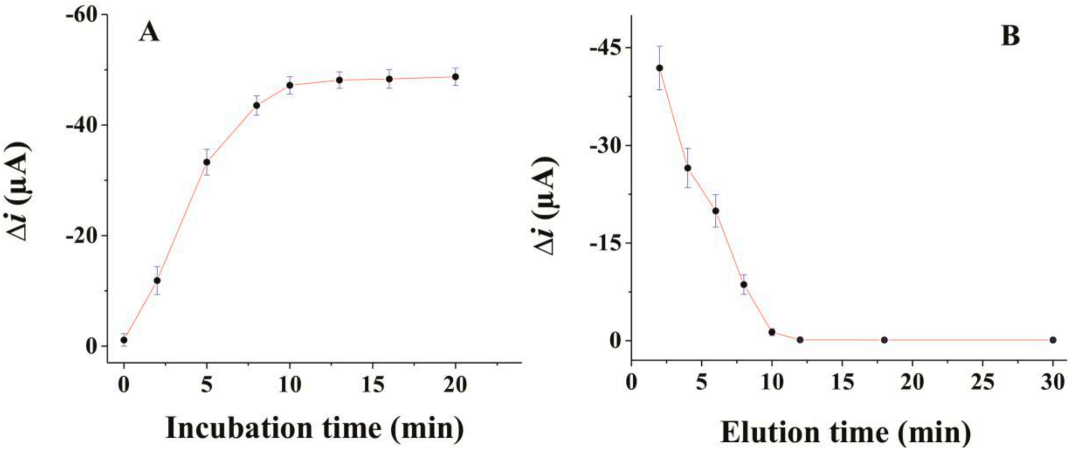

Figure S4. Effects of the (A) incubation time and (B) elution time on the response current from GCE/GO–PtCo@MIPDA. The concentration of TZ was 3.50 μM. Error bars are standard deviations across three repeated experiments.

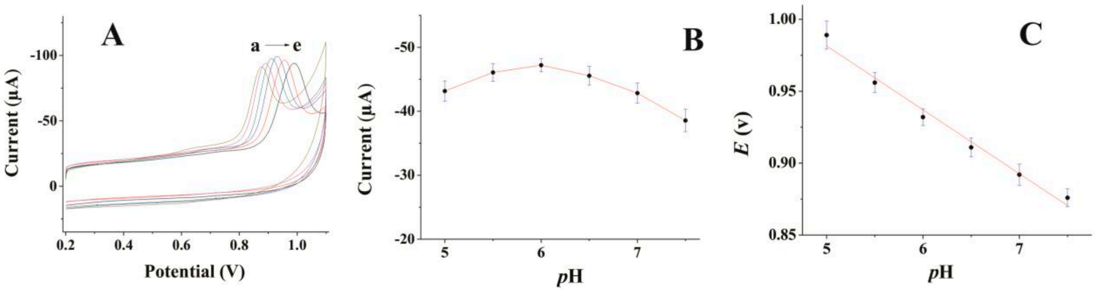

Figure S5. (A) CVs of GCE/GO–PtCo@MIPDA in 0.2 M acetate buffer solutions with 3.50-μM TZ and pHs of (a) 5.0, (b) 5.5, (c) 6.0, (d) 6.5, (e) 7.0, and (f) 7.5. (B) Relationship between the peak current (i_{pa}) of TZ and pH. (C) Relationship between the peak potential of TZ and pH. Scan rate: 0.1 V/s.

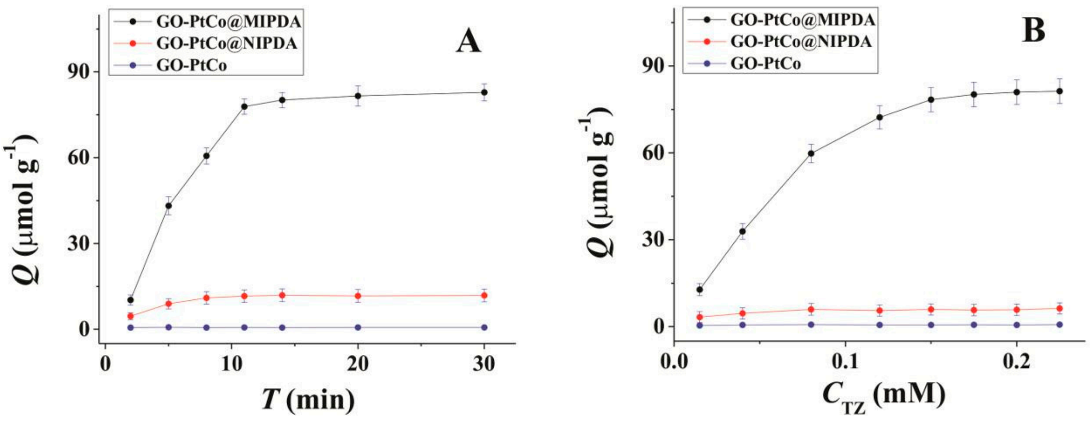

Figure S6. (A) Adsorption kinetics in 0.20 mM TZ solution and (B) adsorption isotherms of various composite materials.

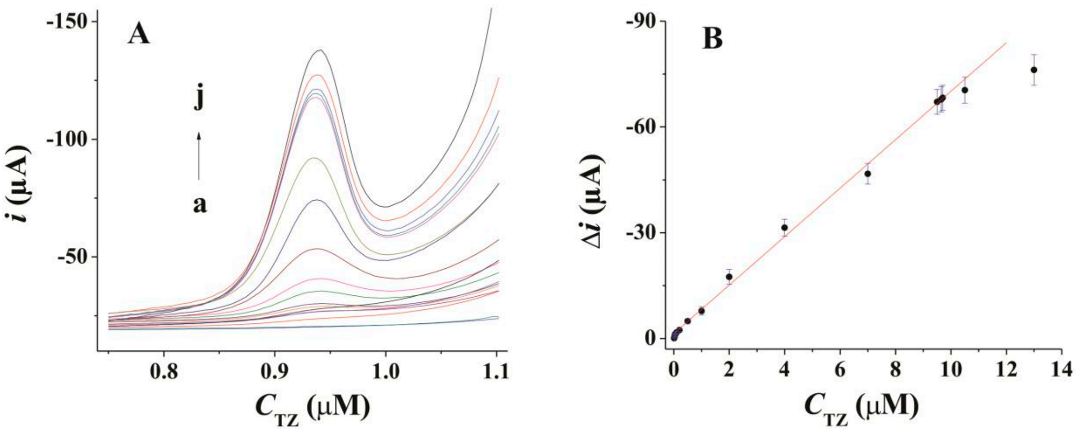

Figure S7. (A) DPVs for GCE/GO–PtCo exposed to TZ concentrations of (from a–j): 0, 0.01, 0.02, 0.03, 0.04, 0.05, 0.07, 0.1, 0.2, 0.5, 1.0, 2.0, 4.0, 7.0, 9.5, 9.65, 9.7, 10.5, and 13.0 μM. (B) Calibration curves of peak current versus TZ concentration at GCE/GO–PtCo. Electrolyte solution: 0.2 M HAc–NaAc (pH = 6.0). Pulse width: 0.2 s. Pulse period: 0.5 s. Amplitude: 0.05 V.

Table S1. Comparison of the analytical performance between the proposed method and graphene/MIP-based electrochemistry methods used in the past decade.

**Electrode (Modifier), Detection Method**	**Linearity (μM)**	**Detection Limit (nM)**	**Recognizing Component, Anti-Interference Ability**	**Recovery from Real Sample**	**Reference**
GN–PTA/GCE, DPV	60~3000 ug/L	30 ug/L	No, good	95~104	[1]
GN–Ni/GCE, SWV	6.24~624 nM	1.08	No, <6%	99.26~100.9	[2]
GN/TiO_2-CPE, SWV	0.02~1018	8	No, <6%	97.41~102.1	[3]
β-CD-PDDA-Gr/GC-RDE, DPV	0.05~20	14.3	No, no selectivity	95.11~103.6	[4]
Graphite/GEC,	5~200 μg/L	2.8	No, no interference	–	[5]
ILRGO-Au/GCE, SWV	7 nM~1.5 uM	0.83	No, <5%	94.7~106.4	[6]

CTAB-GO/MWNT/GCE, DPV	0.03~0.6	5	No, <5%	93~111	[7]
IL-GO-MWCNT/GCE, SWV	0.01~1, 0.02~0.13	7, 0.1	No, good	96.4~99.7	[8]
ERGO-SPCE, DPV	0.02~20	4.5	No, good	80.74~117.59	[9]
ERGO-AuNRs,	0.03~6	8.6	No, no obvious influence	94~108.8	[10]
PDDA-Gr-Pd/GCE, DPV	0.05~8.0	5.0	No, no obvious interference	95~97.65	[11]
GO/MWCNTs/GCE, LSV	0.09~8.0	10	No, highly selective	98~101.75	[12]
N-PC-G/CS/GCE, DPV	0.05~15.0	36	No, good	97~102	[13]
PSSA/Cu(OH)$_2$–Gr/GCE, SWV	0.01~0.6, 0.6~10	8	No, good	–	[14]
TiO$_2$/ErGO/GCE, SDLSV	0.02~20	8	No, good	106.5~112.0	[15]
Gr/PLPA/PGE, DPV	2~100	1.54 μM	No, good	98.71~ 104.44	[16]
rGO/NiBTC/SPCE, DPV	0.075–5.0	50	No, good	94.41~104.73	[17]
PLA-ERGO/GCE，DPV	1~250	250	No, good	98.2~99.6	[18]
TiO$_2$/ErGO/GCE, AdSDPV	0.01–5.0	6	No, good	97.9~108.6	[19]
Pt/CQDs@rGO/SPCE, DPV	0.01~9.3	7.93	No, good	96.0~103.5	[20]
PTH/MWCNT-G(5%)/GCE, SWV	1~100；	250;303	No, good	97.87~106.7	[21]

	250~450				
IL/$NiFe_2O_4$/rGO/CPE, DPV	0.05~30	30	No, good	98~104	[22]
AuNPs/PDDA-Gr/GCE,	0.008~3.00	2.5	No, good	93.5~95.7	[23]
MIP-polypyrrole/GCE, SWV	1~10 nM	1	MIP, –	–	[24]
MIP–MWNTs-IL@PtNPs/GCE, DPV	0.03~5.0, 5.0~20	8	MIP, good	88~108	[25]
MIP-PmDB/PoPD-GCE, DPV	5.0 nM~1.1 µM	3.5	MIP, satisfactory	–	[26]
MIP-acrylamide-CN/GCE, DPV	0.08~1, 1~10	27.4	MIP, good	94.8~101.9	[27]
GCE/GO-PtCo@MIPDA, DPV	0.003~0.180 µM, 0.180~3.950 µM	1.1	MIP, excellent	95.3~105.1	This work

Graphene (GN, Gr), layer-wrapped phosphotungstic acid (PTA), glassy carbon electrode (GCE), differential pulse voltammetry (DPV), square wave voltammetry (SWV), carbon paste electrode (CPE), cyclodextrin (CD), poly(diallyldimethylammonium chloride) (PDDA), glassy carbon-rotating disk electrode (GC-RDE), ionic liquid (IL), Cetryltrimethyl ammoniumbromide (CTAB), multiwalled carbon nanotubes (MWNTs, MWCNTs), multiwalled carbon nanotubes (MWCNTs), electrochemically reduced grapheme oxide (ERGO), screen-printed carbon electrode (SPCE), linear sweep voltammograms (LSV), N-doped graphene natively grown on hierarchical porous carbon (N-PC-G/CS), Poly(5-sulfosalicylic acid) (PSSA), second-order derivative linear sweep voltammetry (SDLSV), poly(l-phenylalanine) (PLPA), pencil graphite electrode (PGE), adsorptive striping differential pulse voltammetric (AdSDPV), carbon quantum dots (CQDs), polythiophene (PTH), molecularly imprinted polymer (MIP).

References

1. Gan, T.; Sun, J.Y.; Cao, S.Q.; Gao, F.X.; Zhang, Y.X.; Yang, Y.Q. One-step electrochemical approach for the preparation of graphene wrapped-phosphotungstic acid hybrid and its application for simultaneous determination of sunset yellow and tartrazine, *Electrochim. Acta,* **2012**, *74*, 151–157.

2. Gan, T.; Sun, J.Y.; Wu, Q.; Jing, Q.S.; Yu, S. Graphene Decorated with Nickel Nanoparticles as a Sensitive Substrate for Simultaneous Determination of Sunset Yellow and Tartrazine in Food Samples, *Electroanal.* **2013**, *25(6)*, 1505–1512.

3. Gan, T.; Sun, J.Y.; Meng, W.; Song, L.; Zhang, Y.X. Electrochemical sensor based on graphene and mesoporous TiO_2 for the simultaneous determination of trace colourants in food, *Food Chem.* **2013**, *141(4)*, 3731–3737.

4. Ye, X.L.; Du, Y.L.; Lu, D.B.; Wang, C.M. Fabrication of cyclodextrin-coated poly (diallyldimethylammonium chloride)-functionalized graphene composite film modified glassy carbon-rotating disk electrode and its application for simultaneous electrochemical determination colorants of sunset yellow and tartrazine, *Anal. Chim. Acta,* **2013**, *779*, 22–34.

5. Song, X.J.; Shi, Z.; Tan, X.H.; Zhang, S.H.; Liu, G.S.; Wu, K.B. One-step solvent exfoliation of graphite to produce a highly-sensitive electrochemical sensor for tartrazine, *Sens. Actuators B: Chem.* **2014**, *197*, 104–108.

6. Wang, M.L.; Zhao, J.W. Facile synthesis of Au supported on ionic liquid functionalized reduced graphene oxide for simultaneous determination of Sunset yellow and Tartrazine in drinks, *Sens. Actuators B: Chem.* **2015**, *216*, 578–585.

7. Yang, Y.J.; Li, W. CTAB Functionalized Graphene Oxide/Multiwalled Carbon Nanotube Composite Modified Electrode for the Simultaneous Determination of Sunset Yellow and Tartrazine[1], *Russ. J. Electrochem.* **2015**, *51(3)*, 218–226.

8. Wang, M.L.; Zhao, J.W. A Facile Method Used for Simultaneous Determination of Ponceau 4R, Allura Red and Tartrazine in Alcoholic Beverages, *J. Electrochem. Soc.* **2015**, *162(6)*, H321–H327.

9. Jampasa, S.; Siangproh, W.; Duangmal, K.; Chailapakul, O. Electrochemically reduced graphene oxide-modified screen-printed carbon electrodes for a simple and highly sensitive electrochemical detection of synthetic colorants in beverages, *Talanta*, **2016**, *160*, 113–124.

10. Deng, K.Q.; Li, C.X.; Li, X.F.; Huang, H.W. Simultaneous detection of sunset yellow and tartrazine using the nanohybrid of gold nanorods decorated graphene oxide, *J. Electroanal. Chem.* **2016**, *780*, 296–302.

11. Yu, L.L.; Zheng, H.J.; Shi, M.X.; Jing, S.S.; Qu, L.B. A Novel Electrochemical Sensor Based on Poly (Diallyldimethylammonium Chloride)-Dispersed Graphene Supported Palladium Nanoparticles for Simultaneous Determination of Sunset Yellow and Tartrazine in Soft Drinks, *Food Anal. Methods,* **2017**, *10(1)*, 200–209.

12. Qiu, X.L.; Lu, L.M.; Leng, J.; Yu, Y.F.; Wang, W.M.; Jiang, M.; Bai, L. An enhanced electrochemical platform based on graphene oxide and multi-walled carbon nanotubes nanocomposite for sensitive determination of Sunset Yellow and Tartrazine, *Food Chem.* **2016**, *190*, 889–895.

13. An, Z.-Z.; Li, Z.; Guo, Y.-Y.; Chen, X.-L.; Zhang, K.-N.; Zhang, D.-X.; Xue, Z.-H.; Zhou, X.-B.; Lu, X.-Q. Preparation of chitosan/N-doped graphene natively grown on hierarchical porous carbon nanocomposite as a sensor platform for determination of tartrazine, *Chinese Chem. Lett.* **2017**, *28(7)*, 1492–1498.

14. Arvand, M.; Gaskarmahalleh, A.A.; Hemmati, S. Enhanced-Oxidation and Highly Sensitive Detection of Tartrazine in Foodstuffs via New Platform Based on Poly(5-Sulfosalicylic Acid)/$Cu(OH)_2$ Nanoparticles, *Food Anal. Methods*, **2017**, *10(7)*, 2241–2251.

15. He, Q.G.; Liu, J.; Liu, X.P.; Li, G.L.; Deng, P.H.; Liang, J.; Chen, D.C. Sensitive and Selective Detection of Tartrazine Based on TiO_2-Electrochemically Reduced Graphene Oxide Composite-Modified Electrodes, *Sensors*, **2018**, *18(6)*, 1911.

16. Tahtaisleyen, S.; Gorduk, O.; Sahin, Y. Electrochemical Determination of Tartrazine Using a Graphene/Poly(L-Phenylalanine) Modified Pencil Graphite Electrode, *Anal. Lett.* **2020**, *53(11)*, 1683–1703.

17. Wu, J.H.; Lee, H.L. Determination of sunset yellow and tartrazine in drinks using screen-printed carbon electrodes modified with reduced graphene oxide and NiBTC frameworks, *Microchem. J.* **2020**, *158,* 105133

18. Wang, P.L.; Liu, X.; Hu, Q.Q.; Gao, H.; Ma, W. Simple and Rapid Determination of Tartrazine Using Poly(l-arginine)/Electrochemically Reduced Graphene Oxide Modified Glassy Carbon Electrode, *Int. J. Electrochem. Sci.* **2020**, *15(9)*, 8901–8912,

19. Qin, Z.R.; Zhang, J.Y.; Liu, Y.; Wu, J.T.; Li, G.L.; Liu, J.; He, Q.G. A Simple but Efficient Voltammetric Sensor for Simultaneous Detection of Tartrazine and Ponceau 4R Based on TiO2/Electro-Reduced Graphene Oxide Nanocomposite, *Chemosensors*, **2020**, *8(3)*, 70.

20. Mehmandoust, M.; Erk, N.; Karaman, O.; Karimi, F.; Bijad, M.; Karaman, C. Three-dimensional porous reduced graphene oxide decorated with carbon quantum dots and platinum nanoparticles for highly selective determination of azo dye compound tartrazine, Food Chem. *Toxicol.* **2021**, *158*, 112698.

21. AL-Refai, H.H.; Ganash, A.A.; Hussein, M.A. Sensitive and selective voltammetric sensor based on polythiophene nanocomposite mixed MWCNT-G for the determination of Tartrazine, *Synthetic Met.* **2021**, *280*, 116875.

22. Darabi, R.; Shabani-Nooshabadi, M. $NiFe_2O_4$-rGO/ionic liquid modified carbon paste electrode: An amplified electrochemical sensitive sensor for determination of Sunset Yellow in the presence of Tartrazine and Allura Red, *Food Chem.* **2021**, *339*, 127841.

23. Wu, T.X.; Wang, Q.; Peng, X.Y.; Guo, Y.J. Facile Synthesis of Gold/Graphene Nanocomposites for Simultaneous Determination of Sunset Yellow and Tartrazine in Soft Drinks, *Electroanal.* **2022**, *34(1)*, 83 –90.

24. Jiang, S.H.; Xu, J.F.; Xu, P.D.; Liu, L.J.; Chen, Y.; Qiao, C.S.; Yang, S.F.; Sha, Z.L.; Zhang, J.K. A NOVEL MOLECULARLY IMPRINTED SENSOR FOR DIRECT TARTRAZINE DETECTION, *Anal. Lett.* **2014**, *47(2)*, 323–330.

25. Zhao, L.J.; Zeng, B.Z.; Zhao, F.Q. Electrochemical determination of tartrazine using a molecularly imprinted polymer – multiwalled carbon nanotubes - ionic liquid supported Pt nanoparticles composite film coated electrode, *Electrochim. Acta*, **2014**, *146*, 611–617.

26. Zhao, X.Y.; Liu, Y.Z.; Zuo, J.J.; Zhang, J.; Zhu, L.; Zhang, J.K. Rapid and sensitive determination of tartrazine using a molecularly imprinted copolymer modified carbon electrode (MIP-PmDB/PoPD-GCE), *J. Electroanal. Chem.* **2017**, *785*, 90–95.

27. Wang, Z.H.; Shan, Y.J.; Xu, L.J.; Wu, G.F.; Lu, X.Q. Development and Application of the Tartrazine Voltametric Sensors Based on Molecularly Imprinting Polymer, *Int. J. Polym. Anal. Ch.* **2017**, *22*, 83–91.
